# A Review on New Frontiers in Drug-Drug Interaction Predictions and Safety Evaluations with In Vitro Cellular Models

**DOI:** 10.3390/pharmaceutics17060747

**Published:** 2025-06-06

**Authors:** Lara Marques, Nuno Vale

**Affiliations:** 1 PerMed Research Group, RISE-Health, Faculty of Medicine, University of Porto, Alameda Professor Hernâni Monteiro, 4200-319 Porto, Portugal; lara.marques2010@hotmail.com; 2 RISE-Health, Department of Community Medicine, Health Information and Decision (MEDCIDS), Faculty of Medicine, University of Porto, Rua Doutor Plácido da Costa, 4200-450 Porto, Portugal; 3 Laboratory of Personalized Medicine, Department of Community Medicine, Health Information and Decision (MEDCIDS), Faculty of Medicine, University of Porto, Rua Doutor Plácido da Costa, 4200-450 Porto, Portugal

**Keywords:** drug-drug interaction, drug metabolism, cytochrome P450, computational modeling, physiologically based pharmacokinetic model, cell cultures, in vitro to in vivo extrapolation

## Abstract

The characterization of a drug’s ADME (absorption, distribution, metabolism, and excretion) profile is crucial for accurately determining its safety and efficacy. The rising prevalence of polypharmacy has significantly increased the risk of drug-drug interactions (DDIs). These interactions can lead to altered drug exposure, potentially compromising efficacy or increasing the risk of adverse drug reactions (ADRs), thereby posing significant clinical and regulatory concerns. Traditional methods for assessing potential DDIs rely heavily on in vitro models, including enzymatic assays and transporter studies. While indispensable, these approaches have inherent limitations in scalability, cost, and ability to predict complex interactions. Recent advancements in analytical technologies, particularly the development of more sophisticated cellular models and computational modeling, have paved the way for more accurate and efficient DDI assessments. Emerging methodologies, such as organoids, physiologically based pharmacokinetic (PBPK) modeling, and artificial intelligence (AI), demonstrate significant potential in this field. A powerful and increasingly adopted approach is the integration of in vitro data with in silico modeling, which can lead to better in vitro-in vivo extrapolation (IVIVE). This review provides a comprehensive overview of both conventional and novel strategies for DDI predictions, highlighting their strengths and limitations. Equipping researchers with a structured framework for selecting optimal methodologies improves safety and efficacy evaluation and regulatory decision-making and deepens the understanding of DDIs.

## 1. Introduction

The accurate determination of safe and effective drug therapy to optimally manage disease depends on a comprehensive understanding of the pharmacokinetics (PKs), pharmacodynamics (PDs), and pharmacogenomics interrelationships of the drug. Regulatory authorities, the European Medicines Agency (EMA), and the Food and Drug Administration (FDA) mandate PK studies as a fundamental requirement in the evaluation of new substances [1,2]. These studies are intended to characterize the processes of absorption, distribution, metabolism, and excretion (ADME) of the drug. Poor PK profiles can cause inconsistent or insufficient drug levels at the target site, leading to variability in clinical outcomes. Notably, metabolic pathways play a key role in determining drug clearance and interindividual differences in PKs, ultimately affecting the clinical efficacy and safety of pharmaceutical compounds [3,4,5].

Following the administration and absorption of a drug, typically those more lipophilic and poorly water-soluble, a series of chemical modifications occur to convert it into one which is more hydrophilic and easily excreted [5,6,7,8]. Drug biotransformation is essential as the lipophilic properties of drugs can prolong their retention in the body, potentially increasing the risk of toxicity [8,9,10]. Phase I reactions involve a variety of chemical processes, including redox reactions, which produce metabolites with new functional groups (-OH, -COOH, -NH_2_, -SH, etc.) that are more polar and reactive than the parent drug. The modified drugs subsequently undergo phase II reactions, where they are conjugated with endogenous molecules by transferase enzymes, such as uridine diphosphate (UDP)-glucuronosyltransferases (UGTs), sulfotransferases (STs), and glutathione S-transferases (GSTs) [5,8]. Cytochrome P450 (CYP) enzymes are key mediators of phase I oxidative metabolism [5,11], playing a crucial role in drug biotransformation and influencing PKs, bioavailability, and therapeutic outcomes [12]. Although the human genome encodes approximately 50 CYP genes, only a small subset is primarily responsible for xenobiotic metabolism, particularly in the liver. Among these, CYP1A2, -2A6, -2B6, -2C9, -2C19, -2D6, -2E1, -3A4, and -3A5 account for the majority of drug metabolism, with CYP3A4 being the most abundant, comprising 30–40% of total CYP protein in the adult liver [5,13,14]. Collectively, CYPs mediate over 90% of enzymatic drug metabolism [6], making them essential determinants of drug action, safety, and resistance. However, their activity varies widely among individuals due to genetic polymorphisms, epigenetic variants, and environmental factors such as age, gender, nutrition, and disease states [8,15]. Additionally, CYP enzymes are highly susceptible to inhibition or induction by concomitant drugs and/or endogenous metabolites, leading to clinically significant drug-drug interaction (DDI), drug–gene interaction (DGI), and drug-drug–gene interaction (DDGI) [8,16].

In recent decades, there has been a significant increase in overall prescription drug use and polypharmacy—the concurrent use of multiple medications—primarily driven by rising life expectancy and the growing prevalence of various comorbidities [17,18]. While polypharmacy may help manage symptoms and prevent disease complications, it is also linked to major concerns such as poor adherence to treatment, adverse drug reactions (ADRs), DDIs, and higher hospitalization rates [19,20,21]. Recent studies highlight a growing number of patients exposed to polypharmacy, particularly among adults over 65 years old [21,22,23,24]. Therefore, the increased risk of DDIs associated with the concurrent use of multiple drugs has become a critical issue in clinical practice [25]. DDIs occur when two or more drugs are concomitantly taken, potentially enhancing or reducing drug efficacy, increasing toxicity, and, in severe cases, leading to life-threatening ADRs or market withdrawal [26,27,28,29]. Recent data demonstrate an increasing prevalence of DDIs, highlighting a significant need for raising awareness among healthcare professionals regarding the causal effect of DDIs in ADR-related hospital admissions [30,31,32]. These interactions are distinguished based on their impact between PK interactions, which involve alterations in drug disposition through the inhibition or induction of metabolizing enzymes or transporters, and PD interactions, which result from agonistic or antagonistic effects at the drug’s therapeutic target [29]. Due to their lower frequency and the singularity of each PD interaction, PK DDIs are more extensively studied [27]. Several reports document cases of early development termination, refusal of regulatory approval, and market withdrawals due to PK DDIs [26,27,29,33]. Consequently, evaluating a drug’s potential for DDIs has become a critical aspect of drug development and has been integrated into the drug discovery and pharmacovigilance processes to mitigate the risks of costly development failures and undesirable therapeutic outcomes [27].

The assessment of a drug’s potential for DDIs involves the following three key steps: (1) identification of the metabolic pathways responsible for the drug’s elimination; (2) determination of the enzymes and transporters involved in its disposition; (3) characterization of how the drug affects the expression and/or activity of such enzymes and transporters. In the early stages of drug development, a range of in vitro tools have been designed to evaluate drug uptake, metabolism, and excretion, as well as to identify potential ADRs [34]. In addition, regulatory authorities have established standardized assays to assess DDI risk. In January 2020, the FDA released the final version of its 2017 draft guidance ‘’In vitro Drug Interaction Studies—Cytochrome P450 Enzyme- and Transporter-Mediated Drug Interactions Guidance for Industry’’ [35], which states that human-derived cell models are the preferred in vitro systems [36,37] due to significant interspecies differences in drug-metabolizing enzymes. A misprediction of the DDI magnitude could raise safety concerns [29]. Therefore, the implementation of reliable methods for the early identification of DDI risk during drug discovery and development is crucial to support informed decision-making [29,38,39].

While several authors have reviewed the general workflow of DDI assessment, listing assay types, methodologies, and appropriate readouts, the variety of in vitro systems available for conducting these studies has received limited attention. This review aims to address that gap by presenting a comprehensive overview of the diverse in vitro models capable of accurately predicting DDIs, distinguishing between well-established conventional models and emerging technologies with significant potential to serve as future alternatives.

## 2. Reaction Phenotyping Study

According to DDI guidelines [35,40], it is essential to evaluate whether a drug serves as a substrate for drug-metabolizing enzymes, particularly CYP enzymes. This is accomplished through phenotyping studies, which are in vitro approaches for identifying the enzymes and metabolic pathways responsible for drug metabolism [41,42,43,44]. In other words, these studies determine the enzymatic kinetics of specific CYP metabolic pathways and quantify their contribution to the total metabolic clearance [26,45]. Since metabolism-mediated DDIs can arise from the competition of co-administered drugs for the same metabolic enzymes, identifying the key contributors to a drug’s metabolism is crucial to estimating its potential as a victim drug [26].

There are four main approaches to reaction phenotyping, which are as follows: (1) using recombinant CYP enzymes expressed in a cellular model; (2) chemical inhibition, which evaluates the effect of a known CYP enzyme inhibitor on the metabolism of the test compound to determine the contribution of the inhibited enzyme; (3) inhibition by antibodies, which examines the impact of a CYP-specific inhibitory antibody on the metabolism of the test compound; and (4) correlation analysis, which determines the involvement of a specific CYP enzyme by analyzing the reaction rates of the test compound and a known CYP enzyme substrate across multiple human liver microsome (HLM) preparations [41,42]. These assays have been continuously optimized over the years, with critical factors to consider, particularly the choice of in vitro experimental systems.

The selection of an appropriate in vitro system should be based on an understanding of the metabolic reactions contributing to the drug’s clearance and the enzymes responsible for catalyzing these reactions [41]. For example, it is not appropriate to use a CYP enzyme system if the test drug is exclusively metabolized by phase II enzymes. Therefore, regulatory authorities recommend a preliminary analysis of the drug’s metabolic stability using hepatocytes and HLMs to distinguish between CYP450 and non-CYP450 metabolic pathways by comparing metabolic differences in incubations with HLMs and hepatocytes. As a general guideline, both HLMs with chemical inhibitors and cells expressing recombinant human enzymes (RHEs) are suitable test systems for phenotyping [45]. Thus, in this initial step, the following three cellular models can be used: primary human hepatocytes (PHHs), HLMs, and RHE-expressing models.

### 2.1. Human Hepatocytes: The Gold Standard Model

PHHs are considered the gold standard experimental model for the preclinical evaluation of human-specific drug properties [3], including metabolic pathways, DDIs, and toxicity, due to their accurate IVIVE correlations [34,36,37,46]. Major regulatory agencies explicitly recommend the use of PHHs for preliminary drug metabolism studies, as these cells provide results most closely resembling in vivo studies [26,29,47]. First isolated approximately 50 years ago [48,49], PHHs are liver-derived cells maintained in culture, preserving their adult phenotype only for a few days [5]. In the initial days of culture, they are fully metabolically competent, expressing most phases I and II drug-metabolizing enzymes, and thus generating a metabolic profile similar to that observed in vivo [50]. Additionally, the use of intact cells, resulting from proper isolation procedures, enables the preservation of the plasma membrane, maintaining active uptake and excretion mechanisms as well as metabolic processes [5,51] and thereby mimicking hepatocyte function in the human liver.

Despite being the closest cellular model to in vivo conditions, their widespread use remains impractical. First, access to PHHs is highly limited as the sole source of these cells is liver tissue obtained from living donors (not post-mortem samples). PHHs are typically isolated from surplus tissue collected during minor liver biopsies or partial hepatectomies performed for medical reasons. Due to ethical reasons, these samples are not collected exclusively for research purposes. A major issue regarding this source of PHHs is that tissue quality may be compromised by the underlying condition requiring medical intervention, such as cancer, cirrhosis, hepatitis, etc., meaning that not all samples are suitable for research use. Another source of liver tissue is donor livers initially intended for transplantation but ultimately deemed unsuitable due to several factors, including fat accumulation, physical damage, pre-existing liver disease, procedural errors during organ retrieval (e.g., suboptimal warm ischemic or cold storage times), or the lack of a compatible recipient. Currently, PHHs are isolated via the perfusion of a three-sided encapsulated liver segment, using a two-step collagenase procedure. Despite continuous efforts to refine PHH isolation protocols, no method achieves a full hepatocyte yield. Typically, isolations yield approximately 5–30 million viable hepatocytes per gram of liver, which is significantly lower than the estimated 300 billion hepatocytes in the entire human liver [46,52,53].

Another challenge is the preservation of hepatocyte morphology and function, which is not entirely achieved despite optimization efforts. While metabolic pathways, enzymatic cofactors, and active gene expression are integrated during the initial days of culture [5,36], several studies report phenotypic instability of hepatocytes. A study analyzing the evolution of CYP mRNA levels, including CYP1A1, -1A2, -2A6, -2B6, -2C9, -2C19, -2D6, -2E1, -3A4, and -3A5, during PHH culture revealed a time-dependent decline. Immediately after hepatocyte isolation, CYP mRNA levels decrease rapidly, dropping to 10–30% of hepatic baseline levels within 4 h of culture [36]. Several factors contribute to this downregulation. Beyond the artificial environment, evidence suggests that the hepatocyte isolation process [5], which involves collagenase digestion and cell culture components, disrupts the expression of CYP-encoding genes [5,36,54,55,56,57,58,59] and their transcription factors. For example, NF-κB, a negative regulator of CYPs during inflammatory responses, can be triggered by disturbances in the hepatocyte microenvironment. PHHs retain only 20–40% of their initial metabolic activity within the first 48 h of culture, indicating that their use as a reliable tool should be restricted to short-term studies [5]. Additionally, culture medium composition, particularly the presence of serum, negatively affects the formation of bile canaliculi, a special hepatocyte morphology, and may lead to excessive fibroblast proliferation a few days after plating [60]. Additionally, factors such as extracellular matrix (EM) integrity, initial cell density and suspension, and drug concentration also influence the normal behavior of PHHs [61,62]. Some researchers suggest that the sandwich culture configuration, in which hepatocytes are placed between two layers of gelled EMs, prolongs hepatic function maintenance [63,64]. This approach is particularly relevant for repeated in vitro assays given the limited lifespan of PHHs. Kaur et al. [61] provide a comprehensive review on aspects related to the isolation and culture of PHHs.

Still regarding the hepatic phenotype, the use of PHHs from different donors in DDI studies introduces substantial variability in results. In fact, regulatory guidance recommends the use of pooled hepatocytes from several donors for inhibition and induction studies to account for the wide interindividual variability in CYP expression observed in humans. Genetic polymorphisms, hormonal status, diet, smoking, alcohol consumption, age, and prior drug exposure [65] contribute to significant batch-to-batch functional variability in hepatocyte preparations [3,66]. Studies by Goméz-Lechón and Sanchez-Quant [59,67] have demonstrated that variations in liver tissue type and the distinct characteristics of liver samples from different donors are closely associated with hepatocyte viability and functionality, particularly the metabolic capacity of CYP enzymes.

Due to the very limited ability of differentiated hepatocytes to proliferate in vitro, PHH culture must be prepared individually from liver tissue, which is already a scarce resource, further complicating their routine use in drug testing. Consequently, long-term storage protocols for PHHs have been developed and optimized, based on a technique called cryopreservation [5,68,69]. Frozen cells retain cellular functionality and the expression of most phases I and II drug-metabolizing enzymes at levels very close to those in fresh hepatocytes. Drug metabolism patterns in hepatocytes before and after cryopreservation indicate both qualitative and quantitative similarities [68,69]. Comparing freshly isolated PHHs to cryopreserved PHHs demonstrates that cryopreservation facilitates experimental reproducibility, enables repeated studies, and allows for the selection of donors with properties best suited to specific experimental objectives [46]. However, cryopreserved PHHs do not permit an unlimited number of viable cells for use. Furthermore, regardless of whether hepatocytes are freshly isolated or cryopreserved they irreversibly lose their hepatic phenotype and proliferative capacity over time [70].

Considering these limitations, the PHH model should be used exclusively for short-term in vitro assays. In vitro DDI studies do not commonly rely on this cellular model, but regulatory guidelines recommend its use as complementary information [35]. PHHs are widely employed in metabolic stability studies since they are not exclusive to the CYP-family biotransformation enzymes but also include other phase I enzymes (e.g., monoamine oxidase, flavin monooxygenase) and phase II enzymes (e.g., UGTs and sulfotransferases [SULTs]).

### 2.2. Human Liver Microsomes

Phenotyping reactions, as previously established, focus on determining the biotransformation enzyme involved in the metabolism of a given drug [71]. Before compound phenotyping, several authors have emphasized the need to define the predominant clearance mechanisms of the drug under investigation [72,73]. If CYP-mediated reactions contribute less than 30% to the drug’s metabolism, the use of HLMs is unnecessary [35]. This is because HLMs primarily contain enzymes from the CYP450 superfamily, with only a limited presence of phase II enzymes.

**Figure 1:**
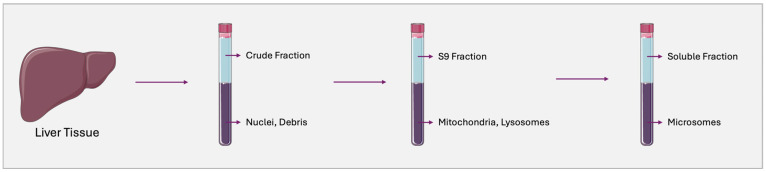
Isolation and preparation of microsomal, S9, and cytosolic fractions routinely employed in drug metabolism studies.

Beyond phenotyping reaction, HLMs are a recommended test system for various in vitro PK studies, including metabolite identification, metabolic stability, enzyme inhibition, and other assessments. HLMs are subcellular fractions derived from the endoplasmatic reticulum of hepatocytes, prepared through liver homogenization followed by differential high-speed centrifugation [73,74,75,76] (Figure 1). They contain membrane-bound phase I enzymes such as CYPs, flavine-containing monooxygenases (FMOs), esterases, amidases, and epoxide hydrolases, as well as phase II enzymes such as UGTs. To sustain the catalytic activity of both phase I and phase II enzymes the addition of exogenous cofactors, including nicotinamide adenine dinucleotide phosphate (NADP) for CYPs and FMOs and uridine diphosphate glucuronic acid (UDPGA) for UGTs, is required [74,77].

These microsomal preparations are commercially available, most commonly sold as pooled HLMs, which consist of microsomes derived from multiple donors. On the one hand, the use of pooled HLMs offers advantages in metabolic studies by accounting for interindividual variability in biotransformation enzyme activity, thereby better approximating the in vivo scenario [74,77]. Notably, several studies have employed gender-specific HLM pools to explore the influence of sex differences on drug metabolism [78]. On the other hand, significant variability in the enzymatic activity of commercial microsomal preparations from batch to batch and across different vendors, stemming from inherent differences in microsome sources and preparation processes, may introduce challenges in data interpretation [73].

Compared to freshly isolated PHHs, HLMs are more readily available, cost-effective, easy to use and store, and offer a simplified system where CYP kinetic measurements are not confounded by other metabolic pathways or cellular uptake processes [74]. Importantly, enzymatic activity is not lost upon freezing, even over extended periods. Studies have documented the retention of CYP enzymatic activity in microsomes, even after multiple freeze–thaw cycles. This thawing restoration flexibility enables researchers to use the same batch across different experiments, facilitating data normalization and comparison while minimizing confounding results introduced by batch-to-batch variability and different vendors [72,73,79,80].

Nevertheless, during in vitro assays compound incubations with HLMs should not exceed 1–2 h and drug concentrations must be carefully optimized beforehand [5,73]. Due to this incubation time limitation, HLMs are not suitable for poorly metabolized compounds. Moreover, they do not fully replicate a physiological environment as compounds primarily metabolized by phase II enzymes cannot be effectively evaluated [35,72,73]. Other factors, including incubation conditions such as ionic strength, pH, and the presence of organic solvents, may also influence microsomal study outcomes [74]. Table 1 presents examples of recent studies investigating PK interactions using HLMs.

### 2.3. Human Liver-Derived Cell Lines: Alternatives

Since the discovery of the in vitro cell culture system in the 19th century, cell culture has been widely employed in biomedical research [87]. Given the inherent challenges associated with the isolation, culture, and maintenance of PHHs, scientists have made significant efforts in recent decades to improve culture systems, enhancing the stability and functionality of hepatic cells [88]. Human liver-derived cell lines theoretically represent an ideal model for drug metabolism screening, DDI studies, and toxicity assessments. These models offer several advantages, including high availability, unlimited lifespan, stable phenotype, ease of handling due to simpler culture conditions, and continuous growth. Comparative gene expression profiles between human liver-derived cells and hepatocytes provide insights into their suitability for specific study designs [89,90,91]. However, one of the major drawbacks of using hepatic cell lines for drug metabolism studies is their low/partial expression of drug-metabolizing enzymes, making them a suboptimal alternative to PHHs. In particular, CYP enzymatic activity is significantly reduced.

Several hypotheses have been proposed to explain the impaired CYP expression observed in hepatoma-derived cell lines. Since these cell lines express sufficient levels of NADPH–cytochrome P450 reductase (CPR), which is responsible for initiating CYP activity, this factor does not contribute to the reduced activity of these enzymes. Instead, more than for post-transcriptional mechanisms, the diminished CYP activity appears to be primarily related to the transcriptional downregulation of CYP genes. A study by Rodríguez-Antona et al. [54] investigated the molecular mechanisms underlying the reduced CYP expression in cultured cells. Their findings indicated that decreased CYP activity may result from reduced expression of activating transcription factors, such as liver-enriched transcription factors (LEFTs), or from higher levels of transcriptional repressors that suppress the expression of CYP-encoding genes. Moreover, alterations in the cellular microenvironment also appear to significantly influence gene expression, as the lack of cell–cell interactions and extracellular matrix (ECM) components can contribute to CYP downregulation in cell culture. Epigenetic mechanisms represent another major factor in gene expression control, including DNA methylation, histone modifications, and non-coding-RNA-associated gene silencing [92,93]. Epigenetic factors are known to regulate the expression of various genes involved in xenobiotic metabolism, particularly those encoding CYP enzymes. Having said that, current research focuses on optimizing hepatic cell lines to exhibit higher CYP enzymatic activity, with several approaches targeting these molecular mechanisms. A review by Donato et al. [3] presents a comparative table of CYP mRNA levels in human hepatoma cell lines and primary hepatocytes. Accordingly, HepG2 and HepaRG cells, two commonly used alternative models, express only 0.03% and 6.8%, respectively, of CYP3A4 mRNA levels compared to hepatocytes, suggesting minimal CYP activity in these cell lines. Based on multiple gene expression profiling studies, unmodified human liver-derived cell lines do not serve as a true alternative model for drug metabolism, particularly DDI studies.

#### 2.3.1. HepG2 Cell Line

HepG2 was the first hepatic cell line developed to exhibit hepatocyte-like characteristics. It was isolated in 1975 from a hepatocellular carcinoma of a 15-year-old Caucasian male with liver cancer (ATCC repository as a human cell line HB 8065) and has been successfully grown in large-scale culture systems [94,95,96,97]. HepG2 cells display some features of normal hepatocytes, such as cell size and shape, with HepG2 cells having a polygonal morphology of 12–19 μm and hepatocytes displaying a cubic shape of approximately 15 μm. Genomic stability and DNA content are also quite similar as HepG2 cells contain ≈ 7.5 pg of genomic DNA (though with reduced stability) while hepatocytes contain ≈ 6 pg of stable genomic DNA [95]. Despite some morphological and functional similarities, HepG2 cells present a poorly developed smooth endoplasmatic reticulum (SER) and contain only half the number of mitochondria typically observed in hepatocytes. This may contribute to their reduced protein synthesis due to the underdeveloped SER and to lower metabolic energy production, which could lead to a decreased availability of cofactors required for catabolism [95,98,99]. Consequently, in addition to the previously reported low expression levels of CYP superfamily genes, enzymatic activity may also be compromised due to these structural limitations. Moreover, the transcriptomic profile of HepG2 cells, demonstrating weak expression of phase I drug-metabolizing enzyme genes, aligns with proteomic findings, where CYP proteins are either absent or found at very low concentrations [100]. For instance, CYP3A4 levels in HepG2 cells are 100–400 times lower than in hepatocytes [100,101]. Contrary to the previously proposed hypothesis for impaired CYP expression, CYP transcripts of CYP1A1, 1A2, 2A6, 2B6, 2C8, 2C9, 2C19, 2D6, 2E1, and 3A4 have been detected in HepG2 cells [102], suggesting the involvement of a potential post-transcriptional mechanism affecting CYP enzyme activity. Similarly, phase II biotransformation enzymes were either detected at very low concentrations or completely absent in HepG2 cells [100,103]. Some studies suggest that UGT, SULT, and GST activities may occur, with GST expression levels being comparable to those observed in hepatocytes. Nevertheless, considering all these factors for phenotyping reactions in DDI screening, where enzymatic activity in drug metabolism must be analyzed and a cell model with basal biotransformation enzyme activity (both phase I and phase II) comparable to hepatocytes is required, unmodified HepG2 cells have limited utility in this type of study. Enzymatic activity is also influenced by culture conditions, cell culture time, and the specific origin of the HepG2 cells [66,104,105,106]. For instance, after approximately 10 passages, HepG2 cells undergo additional alterations, posing a significant obstacle for long-term use [105]. Therefore, optimizing culture conditions is a critical step in ensuring the accuracy and reproducibility of experimental results.

To overcome the low biotransformation capacity of HepG2 cells, several strategies have been developed to enhance the activity of drug-metabolizing enzymes. Approaches such as genetic modification to incorporate one or more drug-metabolizing enzymes, generating CYP-overexpressing cells, and exposure to various compounds have successfully improved metabolic activity. The rationale behind this is to take advantage of the unlimited availability and high proliferative capacity of HepG2 cells while generating metabolically competent cells [89]. Genetic modification is typically performed by transferring human CYP-encoding genes using vectors, such as adenoviral vectors or viral transduction. Adenoviral vectors are particularly effective in transiently inducing CYP expression in HepG2 cells [89,107,108,109,110,111,112,113,114,115,116]. Moreover, they do not produce toxic or mutagenic effects in host cells due to their inability to replicate. However, this also leads to the expression of drug-metabolizing enzymes for only a short period (only a few days) as the viral particles become diluted over subsequent cell divisions. The co-transduction of multiple CYPs has also been successfully employed, allowing the establishment of controlled CYP expression profiles that replicate the metabolic enzyme patterns found in different human populations, such as extensive or poor metabolizers [107,113]. Chen et al. [117] developed a panel of 14 HepG2-derived cell lines, each stably expressing a distinct CYP enzyme, including 1A1, 1A2, 1B1, 2A6, 2C8, 2C9, 2C18, 2C19, 2D6, 2E1, 3A4, 3A5, and 3A7, through lentiviral gene delivery. The authors systematically evaluated these cell lines for CYP expression and enzymatic functionality by analyzing mRNA levels, protein expression, metabolite formation, and long-term culture stability. Their findings demonstrated that the CYP activity of these modified HepG2 cells was superior to that of HepaRG cells and PHHs. While a broad-spectrum CYP panel is ideal, HepG2 cells engineered to overexpress a single CYP enzyme remain highly useful, particularly in phenotyping assays aimed at identifying the primary metabolizing enzyme(s) of a given drug [115,118]. Several studies have also reported the effectiveness of lentiviral transduction for stable gene expression [119,120,121,122]. However, in contrast to adenoviral vectors, which allow for transient high-level expression, lentiviral vectors have a limited capacity in terms of the size of the coding sequences that can be introduced [115].

Another promising strategy involves exposing HepG2 cells to DNA demethylating agents such as 5-aza-2′-deoxycytidine (5-aza-dC), which inhibits DNA methyltransferase 1 (DNMT1). Nakamura et al. [123] explored the effects of zebularine, a cytidine analog and a highly stable second-generation DNA methylation inhibitor. By forming covalent tight bonds between DNMT proteins and zebularine-modified DNA, this compound significantly upregulated the expression of CYP enzymes, with CYP1A1, 2B6, 2C19, and 2E1 showing strong induction, CYP2A6 and 2C9 demonstrating moderate increases, and CYP1A2 and 3A4 displaying only slight upregulation. Consequently, this approach appears to yield HepG2 cells with a more extensive repertoire of functional phase I drug-metabolizing enzymes. In a related study, Ruoß et al. [124] utilized 5-azacytidine and vitamin C to modify the epigenetic status of HepG2 cells, resulting in gene expression patterns closely resembling those of PHHs and leading to enhanced CYP expression and enzymatic activity. Additionally, the use of other compounds, such as bardoxolone methyl [125], phenobarbital, and 3-methylcolanthrene [89], has been reported to selectively increase the expression of certain CYPs.

These studies underscore the diverse strategies available for generating HepG2 cells with enhanced CYP expression. However, while these modified cells can serve as valuable models for phenotyping in DDI studies, the overexpression of a single CYP enzyme may disrupt the natural balance of drug-metabolizing enzymes, as other phase I and II enzymes, as well as drug transporters, may still exhibit low basal expression levels compared to primary hepatocytes [115]. Furthermore, given that HepG2 cells retain some endogenous CYP activity, metabolic findings cannot be solely attributed to the overexpressed enzyme. The reduced expression of transport proteins may limit substrate uptake and metabolite efflux [126]. Altogether, these factors highlight the need for the careful interpretation of experimental results obtained from CYP-overexpressing HepG2 models [126]. Table 2 presents examples of recent studies investigating PK interactions using the HepG2 cell line.

#### 2.3.2. HepaRG Cell Line

HepaRG, a human hepatocellular carcinoma-derived cell line [131,132], is a valuable model for metabolic studies, particularly for phenotyping assays. In 2002, Gripon et al. established this human hepatoma cell line from a female patient with chronic hepatitis C infection and hepatocellular carcinoma [133]. These cells exhibit properties of well-differentiated hepatocytes, displaying liver-specific functions and morphology closely resembling those of human hepatocytes [132,134,135]. When cultured at low density ($2.6\times 10^{6}$ cells/cm^2^), HepaRG cells adopt an undifferentiated, elongated morphology and actively proliferate [134,135,136,137]. They express markers of bipotent hepatic progenitors, which, upon reaching confluence (approximately 10 days after culture initiation), enable differentiation into either hepatocyte or biliary cell lineages depending on the applied culture conditions [131,133,134,135,136,138,139]. Typically, HepaRG cells form hepatocyte colonies surrounded by biliary cells, resulting in a homogenous population of approximately 50–55% hepatocytes and biliary epithelial cells [131,133,134,135,137,140]. In addition, both hepatocyte- and biliary-like cells exhibit a unique property known as transdifferentiation [134,135], whereby a differentiated cell transitions into another cell type. The first cell type consists of granular epithelial cell clusters resembling hepatocytes, while the second type appears more flattened with a clear cytoplasm. Several protocols have been described for obtaining hepatocyte-like cells, often involving the addition of 1–2% of dimethyl sulfoxide (DMSO) and $5\times 10^{-5}$ M hydrocortisone hemisuccinate to the culture medium to induce differentiation. This process results in more granular cells displaying a phenotype similar to human hepatocytes, characterized by the presence of two or more nuclei and functional bile canalicular structures [135,137]. In the differentiated state, HepaRG colonies express and maintain liver-specific functions for a defined period [131,134,140].

A key characteristic that makes this cell line particularly useful for metabolic studies is its ability to express the full range of CYP enzymes, a feature not commonly observed in other human hepatoma-derived cell lines [132,134,138,139,140]. Upon differentiation, HepaRG cells express specific glycolytic enzymes, the liver transcription factor hepatic nuclear factor 4 (HNF4) [131,134], and high levels of mRNA encoding aldolase B [137], which are all markers of mature adult hepatocytes. Notably, aldolase B expression in HepaRG cells represents around 20% of the levels found in freshly isolated human hepatocytes. In contrast, HepG2 cells do not express this hepatic marker and multiple studies have linked the absence of nuclear transcription factors in HepG2 cells to the reduced expression of CYP enzymes.

The hepatocyte-like cell population in the HepaRG cell line exhibits drug-metabolizing enzyme activity for both phase I and phase II metabolism, as well as transporter and nuclear receptors, at levels comparable to those measured in PHHs [131,136,137]. Due to these properties, several authors consider HepaRG cells to possess a metabolic performance similar to that of primary hepatocytes while retaining the proliferative capacity of hepatic cell lines [136]. A whole-genome expression analysis of the HepaRG cell line has revealed gene expression patterns highly similar to those of PHHs and human liver tissues [141]. Specifically, of the 115 genes involved in drug metabolism HepaRG cells exhibit expression levels closely resembling those of PHHs, except for CYP1A2, CYP2A6, CYP2D6, and CYP2E1 which are expressed at lower levels resulting in reduced enzymatic activity. Accordingly, compounds metabolized by phase I xenobiotic-metabolizing enzymes, including CYP2B6, 2C9, 2C19, and 3A4, as well as phase II enzymes, such as UGTs and SULTs, can be readily investigated using this cell line [140]. The high levels of CYP2C9 activity in HepaRG cells are likely attributed to the presence of hydrocortisone in the culture medium as this enzyme is known to be induced by glucocorticoids [137,142]. Conversely, basal CYP2D6 activity is generally at the detection limit, suggesting that HepaRG cells originate from a patient classified as a poor metabolizer for CYP2D6 [137]. The expression of nuclear factors present in HepaRG cells can explain the regulation of drug-metabolizing enzymes in these cells [134,137]. Evidence indicates that HepaRG cells maintain stable metabolic activity for up to 14 days following a two-week differentiation period [131,143,144]. Another study reported sustained CYP enzyme expression for up to one month when cultured in the presence of DMSO [145]. Indeed, enzyme expression levels depend on the duration of confluence and the culture conditions, including the presence of DMSO in the culture medium. The effects of DMSO removal from the culture medium were investigated, observing a significant reduction in CYP enzyme expression levels, particularly CYP3A4, CYP1A2, CYP2B6, and CYP2C9 [134,144,146]. While CYP2C19 activity was less affected, phase II enzyme expression, specifically UGTs and GSTs, also decreased upon DMSO withdrawal [131]. Dubois-Pot-Schneider et al. [147] conducted large-scale analyses of gene expression and histone modifications to determine the role of DMSO exposure in the differentiation of HepaRG cells. DMSO was found to upregulate genes primarily regulated by PXR and peroxisome proliferator-activated receptor (PPAR), as well as histone acetylation. Aleksandrova et al. [148] demonstrated that serum is an essential component for long-term culture maintenance, whereas DMSO is crucial for cell differentiation and the sustained expression of metabolic enzymes. According to their findings, DMSO activates the transcription factor AP-1, which triggers cell cycle arrest and differentiation while regulating nuclear receptors such as PXR and constitutive androstane receptor (CAR). However, other authors argue that DMSO supplementation interferes with metabolic study results, prompting efforts to optimize HepaRg differentiation protocols [149]. Wang et al. [150] reported a DMSO-free hepatic maturation medium that allows rapid (9–12 days) and efficient differentiation using a cocktail of soluble molecules that mimic the in vivo environment. Regarding optimized HepaRG culture methods, two differentiation protocols developed by Sinson-Young et al. [151] and Gripon et al. [133] were evaluated to determine the most suitable model for metabolic and toxicity studies [152]. The Biopredic protocol (developed by Gripon et al.) was identified as the best method for these purposes. New insights have also emerged to develop a fast and convenient protocol for controlling HepaRG cell differentiation. For instance, Li et al. [153] cultured HepaRG cells on polymeric hydrogel substrates, providing a soft-elastic environment that regulated the differentiation process. Therefore, considering all the advantages and limitations of the HepaRG cell line, it seems to be the most suitable human liver-derived model for studying DDIs. Indeed, several recent studies have reported the use of these cells for identifying potential drug interactions [154,155].

#### 2.3.3. BC2 Cell Line

Another hepatoma cell line is the BC2 cell line, which is derived from human hepatocellular carcinoma (HGB) [156,157]. Multiple stable cell lines (B1 to B20) were established following the initial suspension of tumor cells. Among these, a subset of clonally selected cell lines, including BC2, demonstrated the ability to differentiate and remain stable for several weeks post-differentiation without detachment or cell death [156,157]. This cell line exhibits high homogeneity and stability over two years of culture, expressing specific hepatic functions and suggesting its potential utility as a hepatic model. To our knowledge, few studies have investigated this cell line [156,157,158,159]. A study published in 2001 [156] characterized BC2 cells, reporting measurable basal levels of CYP enzymatic activity, including CYP1A1, 1A2, 2A6, 2B6, 2C9, 3A4, and 2E1, as well as of conjugation enzymes such as UGTs and GSTs. Although the authors noted that enzyme levels were approximately 5–10 times lower than those typically observed in PHHs, they emphasized that the detectable activity indicated gene expression. Thus, BC2 cells represent a candidate for genetic modification to upregulated enzymatic activity, potentially enabling their application in metabolic studies. Despite the lack of recent studies documenting the use of this cell line and the need for further research to expand knowledge on its characteristics, the available data suggest its potential utility in DDI studies, similar to other hepatoma-derived cell lines such as HepG2 and HepaRG. A comparative overview of commonly used hepatic cell lines for metabolism studies, including their respective advantages and limitations, is summarized in Table 3.

## 3. Inhibition Study

Following the identification of xenobiotic-metabolizing enzymes involved in a drug’s metabolism, DDI guidelines recommend determining whether the investigational compound is an inhibitor of these enzymes [35]. As part of the assay, the FDA indicates selective inhibitors for specific CYP enzymes (Table 4).

CYP enzymes generally contain both active and allosteric sites, allowing for the binding of multiple ligands that may act as substrates, inhibitors, and/or activators [26]. As a result, inhibition-mediated metabolism is one of the most frequent mechanisms underlying clinically relevant DDIs [218]. In this process, enzymatic activity is reduced due to direct drug–enzyme interactions [219,220], which can be classified into reversible, quasi-reversible, and irreversible inhibition. Alternative classifications exist in the literature; for example, the FDA [35] categorizes mechanisms as reversible or time-dependent inhibition (TDI), which is often referred to as mechanism-based inhibition (MBI).

### 3.1. Reversible Inhibition Study

Regulatory agencies, such as the EMA [221] and FDA [35], mandate that CYP inhibition studies be conducted using in vitro systems. At this stage, the primary enzymes involved in the metabolism of the investigational compound, as identified in prior phenotyping studies, should be analyzed. A compound metabolized by a specific enzyme has the potential to inhibit another substrate metabolized by the same enzyme, effectively shifting its role from a substrate to an inhibitor [222]. Given this consideration, it is essential to evaluate whether the investigational drug inhibits the enzyme responsible for its metabolism.

The inhibition constant K_i_ should be estimated if the drug exhibits inhibitory potential toward metabolizing enzymes [35,221]. Additionally, the type of inhibitory mechanism must be characterized. The following three commonly used methods are employed in reversible inhibition studies: (1) the single-point assay, which utilizes a single concentration to predict IC_50_; (2) IC_50_ determination, which involves a concentration-gradient assay to determine IC_50_ at a fixed substrate concentration; (3) K_i_ determination, which employs a concentration–gradient assay to determine K_i_ across multiple substrate concentrations. Fu et al. [45] provide a detailed study design for conducting these assessments.

#### Mechanisms of CYP Reversible Inhibition

Reversible inhibition is characterized by restoring enzymatic function once the inhibitor dissociates from the enzyme’s active or allosteric site [26]. It can be classified into the following four subtypes: competitive, non-competitive, uncompetitive, and mixed competitive/non-competitive [223]. The duration of reversible inhibition in vivo depends on the drug’s half-life, though the association and dissociation between the enzyme and substrate are often rapid events [26,218,219,220]. The dissociation equilibrium constant (K_i_) describes the extent of inhibition.

Competitive inhibition occurs when the inhibitor and substrate compete for the same binding site on the enzyme, reducing the enzyme’s availability for substrate metabolism [222,223,224]. The degree of competition depends on the relative affinities of both compounds and their local concentrations [222]. Two main scenarios are highly likely to result in clinically relevant DDIs. The first scenario involves the concomitant administration of two substrates with different affinities, where the higher-affinity substrate (perpetrator) can displace the lower-affinity substrate (victim drug), increasing the victim drug’s K_m_ (indicating reduced affinity) and decreasing its metabolism (lower clearance). Reduced clearance can lead to increased systemic exposure, thereby increasing the risk of adverse effects. This mechanism underlies one of the most common types of DDI, as any given enzymatic substrate has the potential to inhibit the metabolism of another substrate metabolized by the same enzyme. The second scenario occurs when two substrates with different affinities are present at highly different concentrations. If the lower-affinity substrate is at a significantly higher concentration than the higher-affinity substrate then it may outcompete the latter and overcome the inhibitory effect.

Non-competitive inhibition occurs when the inhibitor binds to an allosteric site, causing a conformational change in the enzyme that prevents substrate binding at the active site [26,222]. Unlike competitive inhibition, non-competitive inhibitors do not interfere directly with substrate binding; thus K_m_ remains unchanged while V_max_ is reduced, reflecting decreased enzyme efficiency [223,225]. Uncompetitive inhibition, in turn, occurs when the inhibitor binds exclusively to the enzyme–substrate complex, forming an inactive enzyme–substrate–inhibitor complex [223,224]. Unlike non-competitive inhibitors, uncompetitive inhibitors cannot bind to the free enzyme. This inhibition reduces both V_max_ and K_m_ since it decreases the number of functional enzyme–substrate complexes, shifting the reaction toward equilibrium [223]. Mixed inhibition combines features of competitive and non-competitive inhibition. The inhibitor binds to an allosteric site with variable affinity depending on whether the substrate is already bound. As a result, mixed inhibitors decrease V_max_ and may either increase or decrease K_m_, depending on the inhibitory constant (α) [223].

### 3.2. Conventional Models

According to recommendations [45], HLMs should be the first-line in vitro test system for assessing reversible enzyme inhibition. As a subcellular fraction model, HLMs minimize potential interferences from cellular membrane permeability and drug transporters. In addition, they exhibit a strong correlation with in vivo conditions, containing a broad range of phase I and phase II drug-metabolizing enzymes and displaying high enzymatic stability, which ensures the reproducibility of kinetic inhibition studies. This in vitro model has been previously explored in Section 2.2, where some of its limitations were also highlighted. Nevertheless, HLMs are widely used in DDI studies mediated by enzyme inhibition [226,227,228,229,230,231,232].

Regulatory guidelines [35,221] also recommend using pooled human hepatocytes from more than 10 donors and recombinant enzymes in reversible inhibition studies. While evidence strongly supports that PHHs are the most physiologically relevant model for metabolism studies, their inherent limitations hinder their widespread use. The first studies investigating whether DDIs observed in humans could be replicated in human hepatocytes in vitro were conducted by Li et al. [233], where results suggested that PHHs could serve as a useful system for evaluating the DDI potential of various compounds. Subsequently, numerous studies have employed this cellular model for assessing DDIs [227,230,231,232,234].

The challenge of selecting the appropriate donor tissue given the significant interindividual variability in drug-metabolizing enzyme expression [78,235] is addressed by using pooled HLMs or pooled hepatocytes. Pooled hepatocytes are therefore recommended for general DDI studies to account for interindividual variability, whereas single-donor hepatocytes may be useful for personalized medicine studies, such as evaluating CYP enzyme inhibition within a specific genetic background. Moreover, suspended and plated PHHs serve different purposes in inhibition assays. Suspension hepatocytes are more suitable for single-time-point tests, while plated hepatocytes, due to their longer enzymatic activity retention, can be used for assessing inhibition kinetics over time or for repeated exposure studies.

Comparative studies of the intrinsic clearance (Cl_int_) between HLMs and PHHs have been reported, demonstrating that differences in this PKs parameter provide crucial mechanistic insights [236]. When Cl_int_ in PHHs is higher than in HLMs, this may be attributed to active transporter uptake or non-CYP mediated metabolism (e.g., UGTs and reductases). This is because HLM assays require only the NADPH cofactor, and microsomes predominantly contain membrane-bound enzymes rather than soluble enzymes. Conversely, when Cl_int_ in HLMs is higher than in PHHs it may indicate that the drug’s uptake rate into PHHs is slower than its metabolic rate. Notably, Keefer et al. [237] conducted a study investigating the critical factors influencing Cl_int_ and IC_50_ for CYP3A inhibition in HLMs and PHHs. Their findings suggest that passive permeability plays a critical role in enzymatic inhibition in PHHs, where slow passive permeability may limit the inhibition mechanism. This explains the lower inhibition metrics in PHHs compared to HLMs. In such cases, microsomes may serve as a more predictive model of in vivo clearance than hepatocytes.

Other cellular models are equally useful for assessing reversible enzymatic inhibition, including RHE-expression systems. These models involve the heterologous expression of individual CYP isoforms in host systems such as bacteria (*Escherichia coli*), yeast (*Saccharomyces cerevisiae*), or mammalian cells (baculovirus-infected insect cells) [238]. Recombinant CYP assays offer a highly controlled environment, enabling direct comparison of the inhibitory potential of test compounds across specific CYP enzymes [3]. A major advantage of these systems is the capacity to investigate the inhibition of a single isoform without interference from other metabolic enzymes, thereby avoiding cross-reactivity and off-target metabolic interactions. Moreover, recombinant CYP enzymes require only an NADPH-regenerating system to induce enzymatic activity, which simplifies the experimental setup and eliminates confounding factors such as cellular transporters or phase II metabolic enzymes. As previously mentioned, genetically modified cell lines engineered to express only one CYP enzyme have been shown to yield enzymatic activity data comparable to, or in some cases exceeding, those obtained with gold standard methods.

However, results obtained from recombinant CYP assays must be interpreted with caution. These systems do not fully replicate the physiological cellular environment, which may alter enzyme kinetics, stability, and inhibitor binding affinity. Consequently, while recombinant CYPs offer high specificity, they fall short in capturing the complexity of the microsomal context, where multiple enzymes and cofactors interact and influence metabolic processes [239]. As a result, despite their utility in early screening phases, findings from RHE-expressing models typically require further validation using more physiologically relevant systems, such as HLMs or PHHs, to ensure accurate prediction of in vivo DDI risk.

Liver S9 fractions, derived from tissue homogenates through successive centrifugations, represent a composite in vitro system that contains both microsomal and cytosolic components. This configuration enables the simultaneous assessment of phase I and phase II metabolic pathways, including their respective cofactors, thereby providing a more comprehensive simulation of hepatic metabolism than isolated microsomal or cytosolic preparations. Due to this integrative enzymatic profile, S9 fractions allow for the characterization of a drug’s full metabolic fate, encompassing both CYP-mediated and non-CYP-mediated biotransformation. Although S9 fractions offer a more complete enzymatic representation compared to microsomes or cytosol alone they do not preserve the intact cellular architecture as they lack plasma membranes and, consequently, membrane-bound transporters. This absence limits their ability to fully replicate the physiological transport process, which may be relevant in certain metabolic or inhibitory pathways [240]. While regulatory agencies do not specifically endorse S9 fractions as an in vitro model for inhibition studies, their capacity to concurrently evaluate CYP and non-CYP metabolism, coupled with their relatively low cost and technical simplicity, renders them a valuable tool in early-phase screening. As such, S9 fractions remain a pragmatic and informative alternative for preliminary inhibition assessments.

### 3.3. Emergent Models

The pharmaceutical industry has increasingly prioritized the early prediction and identification of potential enzyme inhibitors during the initial phases of drug development. This proactive approach aims to prevent the substantial financial losses associated with the withdrawal of candidate compounds at later and more costly stages [241]. In this context, expanding the repertoire of reliable and scalable in vitro models is crucial for improving the predictability of preclinical findings and their translatability to human physiology.

To perform accurate metabolic screenings, in vitro human hepatic models must exhibit both morphological and functional properties that closely reflect native liver physiology [242,243]. However, conventional two-dimensional (2D) cell culture systems traditionally employed in such studies lack essential liver-specific functions, gene expression profiles, and cell–cell and cell–extracellular matrix (ECM) interactions [244,245,246,247]. Consequently, they fail to recapitulate the structural complexity and heterogeneity of the hepatic microenvironment. Monolayer 2D cultures, being confined to a horizontal plane, do not permit dynamic spatial interactions and are uniformly exposed to culture medium, lacking the physiological gradients of soluble factors, nutrients, oxygen, and waste products that hepatic cells encounter in vivo. As a result, 2D cultures frequently fall short in mimicking the metabolic outcomes of drugs observed in humans [248]. Nonetheless, due to their simplicity, ease of handling, and cost-effectiveness, they remain the preferred in vitro models in many laboratories.

**Figure 2:**
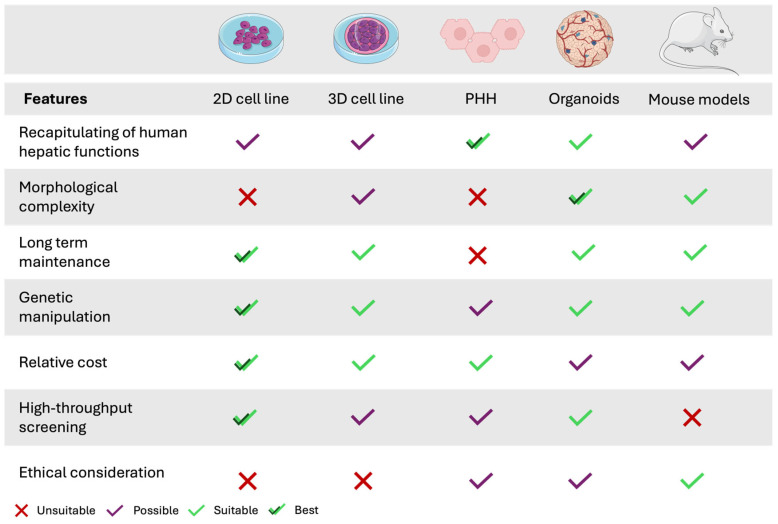
Different cell model systems. Abbreviations: 2D, two-dimensions; 3D, three-dimensions; PHH, primary human hepatocytes. This figure was partly generated using SMART—Servier Medical Art, licensed under CC BY 4.0 (https://creativecommons.org/licenses/by/4.0/). and BioRender. Available online: https://smart.servier.com and https://www.biorender.com (accessed on 22 April 2025).

Animal models, while offering a more comprehensive physiological context than human-derived hepatic cell lines, present inherent limitations. Interspecies differences in hepatic physiology, drug metabolism, and phenotypic responses prevent the accurate extrapolation of preclinical data to humans [242,247]. Consequently, conventional methods often struggle to yield reliable insights into human-specific metabolism and toxicity. In this context, the development of non-animal alternatives, aligned with the 3Rs principle (replace, reduce, and refine animal use), has gained significant impetus over recent years [249,250]. Figure 2 displays the cellular model systems available for in vitro metabolism studies.

Three-dimensional (3D) culture systems have emerged as a promising strategy to bridge the gap in existing cell-based models [251]. A landmark advancement in this field was the development of organoids, enabled by the progress in stem cell technologies in the 1980s [252]. Organoids are 3D cell aggregates derived from stem cells that undergo self-organization, self-renewal, and differentiation into tissue-like structures that resemble the architecture and function of their organ of origin [253]. According to Lancaster and Knoblich [254] and Huch and Koo [255], organoids are defined as in vitro 3D cellular clusters derived from tissue-resident progenitor or stem cells, embryonic stem cells (ESCs), or induced pluripotent stem cells (iPSCs), which recapitulate key features of the source tissue’s functionality. Emerging evidence demonstrates that stem cells can generate hepatic organoids using 3D culture techniques [247], making them one of the most promising in vitro models for drug metabolism studies. Notably, Huch et al. [256] argue that human liver organoids display hepatic functions more comparable to in vivo tissue than conventional hepatic cell lines such as HepG2. Other authors, however, have reported that these emerging models do not yet achieve the same level of hepatic function as PHHs [257]. The truth is that numerous efforts have been made to develop functional hepatocyte-like cells from hepatic organoids, and several studies have been published demonstrating different organoid generation protocols, each yielding different outcomes in terms of hepatic functionality. Nevertheless, systematic comparative studies between organoid-based models and established systems such as PHHs and HLMs are still needed to ensure predictability in metabolic studies.

**Figure 3:**
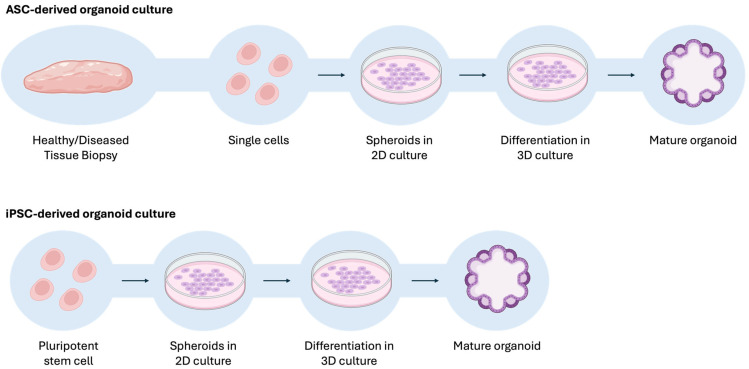
Organoids generation from ASCs and iPSCs. ASC-derived organoids are generated from healthy or tumor biopsies; the tissue is dissociated into a single-cell suspension and embedded in an ECM; a tissue-specific growth factor-enriched medium is added and refreshed regularly to support organoid expansion. iPSC-derived organoids begin as 2D cultures, which are induced to form aggregates or spheroids; and these structures are then embedded in an ECM and matured using tissue-specific growth factor-enriched medium.

Hepatic organoids can be derived from a variety of stem cell sources, including liver progenitor cells, hESCs, adult stem cells (ASCs), and iPSCs [258,259]. iPSC-derived organoids are generated from reprogrammed iPSCs, which possess the ability to differentiate into various cell types representing multiple organ systems [260]. In contrast, ASC-derived organoids (also referred to as patient-derived organoids, PDOs) are generated directly from the dissociation of healthy or diseased tissue and subsequently cultured with tissue-specific growth factors, thereby allowing a more accurate recapitulation of the original tissue phenotype [261]. These cells are then directed to differentiate into hepatic tissues using specific cocktails of growth factors [262], resulting in a cell niche that preserves the phenotype of the original tissue. The establishment of more physiological, biochemical, and biomechanical microenvironments, through 3D techniques, can positively influence cellular proliferation, differentiation, migration, mechanotransduction, and cell survival [248,263]. During culture, liver organoids are capable of maintaining both structural integrity and function over extended periods, with many morphological and physiological characteristics preserved even after multiple passages [247,264]. From a technical standpoint, the generation of iPSC-derived organoids is more complex and time-consuming than that of ASC-derived organoids (Figure 3). This is primarily due to the pluripotent nature of iPSCs, which must first be directed toward the appropriate germ layer (ectoderm, mesoderm, or endoderm) before initiating differentiation into the target tissue or organ. In contrast, ASCs are already committed to a specific organ lineage, simplifying their protocol generation [259]. Indeed, the critical phase in the formation of iPSC-derived organoids is the commitment of cells to the desired germ layer, achieved through the application of specific inductive signaling factors such as wingless-type mouse mammary tumor virus integration site family (WNT), transforming growth factor-beta (TGF-β), and fibroblast growth factor (FGF). These signals guide the cells to adopt characteristics of the intended tissue or organ [265,266]. For this reason, iPSC-derived organoids often display an embryonic-like phenotype, which can influence the expression and activity of drug-metabolizing enzymes. In contrast, ASC-derived hepatic organoids, obtained directly from liver tissues, tend to retain more mature hepatic functions and may, therefore, represent a model that mimics more closely in vivo enzymatic activity.

In terms of metabolic capacity, particularly the expression and activity of CYP enzymes, liver organoids are believed to closely resemble liver tissues. However, as far as current evidence indicates, further studies are warranted to comprehensively evaluate CYP expression and enzymatic activity in organoids compared to other hepatic models, such as HepG2 and HepaRG cell lines. Park et al. [267] investigated drug metabolism and toxicity using intestinal and hepatic organoids derived from mice and concluded that these models closely reflect in vivo observations. Similarly, Bouwmeester et al. [242] demonstrated that hepatocyte-like organoids (intrahepatic cholangiocytes formed by ASCs) displayed CYP3A4 expression levels comparable to those of PHHs and HepaRG cells, whereas CYP2B6 and CYP2D6 levels were lower. These findings underscore the need for further exploration of hepatic organoids as in vitro models for metabolic studies. Their potential to combine physiological relevance with scalability and reproducibility positions them as powerful tools in the future of drug development and personalized medicine.

In addition to interspecies differences limiting the applicability of animal models in drug metabolism and toxicity studies, intraspecies variability can also mask xenobiotic responses. Interindividual variability within the human population often leads to significant differences in drug efficacy and toxicity among individuals. In this context, iPSC-derived organoids have emerged as a promising alternative to detect such variability and to support the development of therapies tailored to patient-specific characteristics.

Recent advances have enabled the generation of patient-derived organoids (PDOs) [268], including hepatic organoids derived from iPSCs. Mun et al. [269] reported a robust and reproducible method for generating mature and functional hepatic organoids from iPSCs. Currently, in vitro differentiation protocols exist to differentiate hepatocyte-like cells (HLCs) from iPSCs [270]. In brief, somatic cells from individual donors are collected and reprogrammed into human iPSCs (hiPSCs). These hiPSCs are then differentiated into definitive endoderm using a cocktail of growth factors (such as activin A and WNT), followed by further differentiation into hepatic progenitor cells. This process typically spans approximately 25 days, at which point HLCs are obtained which have a high potential for use in metabolism and toxicity studies. Nevertheless, the expression of certain CYP genes remains suboptimal in HLCs. To ensure that in vitro results accurately reflect in vivo outcomes, upregulation of specific CYP enzymes is required, necessitating further optimization of differentiation protocols. Shinozawa et al. [271] successfully developed an organoid-based assay with multiplexed readouts to evaluate cell viability, cholestatic injury, and mitochondrial toxicity, achieving high predictive accuracy across 238 marketed drugs. More recently, Kim et al. [272] described a cultivation optimization strategy that significantly enhanced both the expression and activity of CYP enzymes in hepatic organoids, particularly CYP3A4, CYP2C9, and CYP2C19.

Although organoid technology represents one of the most promising tools in the biomedical field, several *speed bumps* need to be addressed for future research [273,274]. First, current organoid models are relatively simple and lack a vascular system. When organoids reach a certain size then cells in the core are unable to receive sufficient nutrients and oxygen and the removal of metabolic waste becomes challenging. Second, the immune microenvironment is not fully recapitulated in current organoid models. The absence of physiological immune–cellular interactions limits the ability of these systems to accurately mimic drug responses and therapeutic effects observed in vivo. Third, the ECM, a complex and hierarchical network essential for organoid culture, does not fully meet the structural and biochemical needs of organoid development. Current ECM materials often lack key components required for optimal growth, reproducibility, and large-scale production of organoids. Additionally, the high cost and technical expertise required for the development and maintenance of these advanced cellular models also present barriers to broader adoption and application. To date, the application of hepatic organoids in DDI studies remains limited.

## 4. Induction Study

The induction of drug-metabolizing enzymes can increase the clearance of drugs, resulting in lower plasma concentrations and potentially reduced pharmacological efficacy. Additionally, enzyme induction may enhance the activation of prodrugs, thereby altering the PKs of the parent compound, or even increase the risk of toxicity due to the formation of reactive metabolites as a consequence of increased metabolic rates. Enzyme induction, often triggered by the co-administration of multiple drugs, is characterized by an increase in the expression and activity of enzymes following exposure to xenobiotic (or endogenous) inducers. This process is comparatively slower than enzymatic inhibition as it involves the upregulation of enzyme biosynthesis [26,45].

Enzyme induction is mediated through nuclear receptor pathways, primarily involving the aryl hydrocarbon receptor (AhR), pregnane X receptor (PXR), and constitutive androstane receptor (CAR), which are commonly associated with the regulation of CYP1A2, CYP3A4, and CYP2B6, respectively. Briefly, the drug activates one of these nuclear receptors, which subsequently induces the transcriptional upregulation of target genes encoding drug-metabolizing enzymes [26,45].

Regulatory guidelines identify PHHs and human-derived cell lines, such as HepaRG, as the most suitable in vitro systems for evaluating the induction potential of candidate drugs, given their close approximation to the in vivo hepatic environment [35]. Upon differentiation, HepaRG cells express a wide range of CYP enzymes and their regulatory receptors at levels comparable to freshly isolated human hepatocytes, supporting their predictive utility for in vivo enzyme induction.

The initial assessment of a drug’s induction potential should focus on CYP1A2, CYP2B6, and CYP3A4/5. If no induction of CYP3A4/5 is observed then further evaluation of CYP2C8, CYP2C9, and CYP2C19 is generally not required since both CYP3A4/5 and the CYP2C subfamily are regulated via PXR activation. If CYP3A4/5 is otherwise detected then subsequent assessment of the CYP2C isoforms is necessary [35]. These assays require the inclusion of inducers of enzymes from the CYP superfamily, which are presented in Table 5.

Given the large number of genes involved in the expression of drug-metabolizing enzymes, the traditional view of DDIs must be expanded to also account for genetic variability [275]. DDIs may be triggered not only by the inhibition or induction of metabolic enzymes by co-administered drugs but also by loss-of-function (LOF) or gain-of-function (GOF) genetic variants that alter enzyme activity and, consequently, the metabolism of victim drugs. In some cases, genetic variation and the perpetrator drug act in concert to modulate the metabolic pathways of the victim drug, significantly impacting its plasma concentration. Additionally, phenoconversion may occur, whereby the effect of the interacting drug and the genotype are opposing, resulting in a temporary shift in phenotype. In this context, in vitro cellular models can serve as controlled platforms to dissect such complex interactions. For example, the interaction between clopidogrel and CYP2C19 inhibitors (e.g., omeprazole) is highly influenced by CYP2C19 polymorphisms [276]. LOF alleles impair the bioactivation of clopidogrel, an effect that can be further exacerbated by the co-administration of enzyme inhibitors. Genetically modified hepatic cell lines carrying specific genotypes can be employed to model these scenarios. Phenoconversion related data can likewise be generated in vitro. For instance, inflammatory cytokines (e.g., inteleukin-6) are known to suppress CYP3A4 expression, effectively converting a normal metabolizer into a poor metabolizer phenotype [277]. This can be modeled by exposing hepatocyte cultures to pro-inflammatory mediators. Therefore, even these complex interactions can be evaluated using in vitro models, generating mechanistic data that may be leveraged for complementary applications including pharmacometric studies.

## 5. Future Perspectives: Hybrid In Vitro–In Silico Approaches

In vitro assays represent a crucial component in detecting DDIs, particularly during the drug development and regulatory approval phases. However, several limitations are inherent to laboratory-based DDI studies, including high associated costs, the challenge of clinically recognizing DDIs, dose-dependent interaction, the regulatory approval framework, and demographic and genetic variability among individuals [278,279,280]. Moreover, despite the functional similarities among different models, notable differences in kinetic outcomes are frequently observed across various in vitro systems. Empirical data often diverge between these systems, even under standardized conditions and substrate concentrations where ideally similar results would be expected. These discrepancies can be attributed to factors such as differences in protein content and enzyme ratios, which, as discussed throughout this manuscript, vary significantly between in vitro models. Such inconsistencies highlight the need for integrated approaches to improve the reliability of in vitro-to-in vivo extrapolation (IVIVE) [281].

In this context, computational approaches, or in silico pharmacology, have recently emerged as powerful frameworks for accelerating the identification and prediction of DDIs (Table 6). These approaches significantly reduce societal and financial burdens by leveraging modeling and simulation (M&S) strategies. The growing availability of large-scale drug-related datasets, such as electronic health records (EHRs) and public pharmacological databases, supports the development of increasingly robust computational models. These models are data-driven, and their accuracy and reliability are highly dependent on the quality and integration of the input data [279].

Among the diverse types of drug-related information that can be integrated into these models, data generated from in vitro systems, such as IC_50_, K_i_, and Cl_int_, serve as essential inputs for the building of physiologically based pharmacokinetic (PBPK) and quantitative systems pharmacology (QSP) models. DDI predictions from in vitro data can be conducted using either static mechanistic models or dynamic models [286]. Static models assume constant concentrations of the perpetrator drug and incorporate potency measurements and sensitivity parameters for the victim drug toward the affected mechanism. These models are typically employed in the early stages of drug development to estimate the DDI potential between known victim and perpetrator drugs, especially those involving major CYP enzymes. However, static models are not usually applied for quantitative DDI predictions, but rather they serve as early-warning tools for identifying potential changes in drug exposure (e.g., area under the curve, AUC). In contrast, dynamic models, such as PBPK models, consist of multiple interconnected physiological compartments representing various tissues of the human body. Unlike static models, PBPK models incorporate time-dependent concentrations of both perpetrator and victim drugs across organs and systemic circulation, allowing for more refined predictions [286,287,288]. In simulation platforms like Simcyp^®^ and GastroPlus^®^, in vitro data—often generated from various cellular models such as hepatocytes, HLMs, or recombinant enzyme systems—can be seamlessly integrated [289]. Moreover, interindividual variability can be incorporated, enabling the identification of patient subgroups at elevated DDI risk [290].

Computational approaches to DDI prediction can also be categorized into the following three methodological groups: (i) similarity-based methods, which assess similarity scores between drugs based on structural features, gene expression profiles, and pharmacological properties; (ii) network-based methods, which utilize drug similarity networks and PPI networks to infer potential interactions; (iii) machine-learning (ML) approaches, which integrate diverse data sources to capture various aspects of drug behavior, including adverse effects, target similarity, and signaling pathways. In particular, ML and artificial intelligence (AI) approaches have gained significant attention due to their high accuracy and efficiency in predicting potential DDIs through the analysis of complex datasets [286,291]. AI models can incorporate diverse data types, including molecular structures, biological networks, clinical data, and in vitro results, thereby enabling more precise and extrapolatable DDI predictions [292]. These computational strategies not only reduce the time and cost associated with experimental approaches but also enhance the scale, accuracy, and translational potential of DDI predictions [286].

## 6. Conclusions

The accurate prediction of drug metabolism and the risk of DDIs is fundamental to the development of safe and effective drugs. As polypharmacy becomes increasingly prevalent so does the need for reliable methodologies capable of anticipating and characterizing clinically significant DDIs. Conventional in vitro models, including PHHs and hepatic cell lines, have significantly contributed to our understanding of xenobiotic metabolism. However, their intrinsic limitations, such as restricted lifespan, low CYP450 expression, and absence of interindividual variability, highlight the need for more physiologically relevant models.

Recent breakthroughs in hiPSC technology and 3D culture systems have paved the way for a new generation of in vitro models. These systems not only allow for the modeling of complex hepatic functions but also provide a powerful platform for advancing personalized medicine. Despite these advances, further efforts are required to refine the differentiation protocols and improve the functional maturity of these cellular models. Advancing the field requires collaborative efforts across academia, industry, and regulatory agencies to validate and adopt next-generation models that better reflect human physiology and variability. In this context, one of the major challenges is the lack of standardized and harmonized protocols for cell culture and differentiation. Variations in culture conditions, such as the ECM used, growth factor combinations, and differentiation timelines, can significantly affect the reproducibility and functional performance of these systems. This variability hinders cross-study comparisons and limits the broader application of organoid systems in pharmacological research. Establishing consensus guidelines and minimal quality criteria for in vitro model generation and characterization is therefore essential.

## Figures and Tables

**Table 1:** Summary of selected studies investigating DDIs using HLMs.

Study Example	Description
Yu et al. [81]	A study between donepezil and tadalafil, both primarily metabolized by CYP3A, using pooled HLMs. Tadalafil was found to concentration-dependently inhibit donepezil metabolism.
Liu et al. [82]	The DDI potential of vicagrel was investigated using pooled HLMs and PBPK modeling. Vicagrel potently inhibited CYP2B6 and CYP2C19 and showed mixed-type and noncompetitive inhibition for bupropion and S-mephenytoin metabolism, respectively. PBPK simulations suggest vicagrel poses low DDI risk with these substrates.
Yang et al. [83]	Potential PK interactions between bicyclol and commonly co-administered agents were evaluated using rat liver microsomes (RLMs) and HLMs. Bicyclol was notably inhibited by pioglitazone, fenofibrate, tacrolimus, and cyclosporin A. However, as the selected inhibitory drug concentrations in vitro exceeded clinically relevant levels and maximum inhibition remained below 50% the risk of clinically meaningful DDIs involving bicyclol in humans appears low.
Li et al. [84]	The study assessed the impact of carvedilol on the metabolism of bedaquiline using in vitro systems, including RLMs and HLMs, a recombinant CYP3A4 system, and in vivo rat models. Their findings suggest that carvedilol can inhibit bedaquiline metabolism.
Faison et al. [85]	Evaluation of the PKs and safety of dordaviprone (ONC201), a novel antitumor agent, when administered alone and with itraconazole. It represents an integrated approach combining in vitro experiments with clinical investigation. In vitro assays using HLMs and rCYP enzymes demonstrated that CYP3A4 is the primary enzyme involved in dordaviprone metabolism. In healthy participants, co-administration with itraconazole significantly increased dordaviprone maximum plasma concentration and area under the curve, confirming a CYP3A4-mediated drug interaction.
Jaisupa et al. [86]	The study investigated the metabolic interaction between cannabidiol (CBD) and commonly co-administered antiseizure medications, as well as the influence of intermediate-activity CYP2C19 genotypes. Using pooled HLMs, the intrinsic clearance of CBD was reduced when combined with antiseizure medications. No significant difference in CBD metabolism was observed between HLMs from CYP2C19*1/*2 and *1/*4 donors.

**Table 2:** Summary of selected studies investigating DDIs using the HepG2 cell line.

Study Example	Description
Xue Li [127]	The influence of CYP2C19 genetic polymorphism on the DDI between voriconazole and omeprazole was investigated using lentivirus-engineered HepG2 cell lines expressing either CYP2C19*1 or CYP2C19*2. Although omeprazole inhibited voriconazole in both genotypes, the IC_50_ for CYP2C19*1 was slightly lower, suggesting a marginally stronger inhibitory effect.
Xun et al. [128]	This study examined the PK interaction between voriconazole and atorvastatin using a comprehensive approach that included clinical data, in vivo experiments in rat models, and in vitro models. Among the in vitro systems, HepG2 cells were employed to assess the metabolic profile of atorvastatin in the presence of voriconazole.
Sager et al. [129]	Using HepG2 cells and plated PHHs it was demonstrated that bupropion and its metabolites significantly downregulate CYP2D6 mRNA expression in a concentration-dependent manner.
Cui et al. [130]	The effects of berberine on lovastatin PKs were analyzed using both in vivo (rats) and in vitro (HepG2 cells) models. Berberine pretreatment significantly decreased lovastatin plasma exposure in rats, indicating enhanced metabolism. Correspondingly, berberine induced increased metabolic activity and altered kinetic parameters of lovastatin in HepG2 cells.

**Table 3:** List of advantages and disadvantages and examples of studies of the different human liver-derived cell lines.

Cell Line	Origin	Advantages	Disadvantages	Applications
HepG2	Human hepatoblastoma	- Easy to culture - Commonly used in toxicity screening - Stable growth	- Very low expression of major CYP450 enzymes - Poor metabolic capacity	[127,130,160]
HepaRG	Human hepatocellular carcinoma	- High CYP450 expression (especially CYP3A4, and CYP1A2) - Good model for both metabolism and toxicity - Closer to PHHs after differentiation	- Requires DMSO for full differentiation - Slow proliferation - Limited availability	[155,161]
BC2	Human hepatoblastoma	- Retains some liver-specific functions - Improved over parental HepG2	- Limited use - Low CYP expression compared to PHHs	[156]
Huh-7	Human hepatocellular carcinoma	- Widely available - Easy to transfect and manipulate genetically	- Low CYP expression - Immature hepatic phenotype	-
PH5CH	Immortalized human fetal hepatocytes	- High proliferation - Potential for metabolic studies	- Fetal origin limits maturity - CYP activity remains low	-
Upcyte	Genetically modified human hepatocytes	- Extended lifespan - Moderate CYP450 activity - Reproducible performance	- Lower CYP expression than fresh PHHs - Limited commercial sources	[162]
PLC	Human hepatocellular carcinoma	- Produces α-fetoprotein - Can be used in hepatotoxicity studies	- Very low metabolic capacity - Poor CYP450 expression	[163]
SNU-182	Human hepatocellular carcinoma	- Tumor-derived, with some liver-like features - Responsive to certain drug stimuli	- Limited CYP expression - Less characterized for metabolic studies	-
SNU-449	Human hepatocellular carcinoma	- Good for studying drug resistance and cancer metabolism	- Poor metabolic function - Low CYP expression	-
Hep3B	Human hepatocellular carcinoma	- Can produce liver proteins - Useful in some toxicity screens	- No expression of p53 - Very low CYP activity	[164]
THLE-2	Immortalized human normal liver epithelial cells	- Non-cancerous origin - Represents normal liver epithelium - Useful for toxicity studies	- Minimal CYP450 activity - Immature hepatic function	[165]
THLE-3	Immortalized normal liver cells	- Slightly improved metabolic activity over THLE-2 - Useful for toxicity studies	- Limited in DDI prediction	-

**Table 4:** The FDA recommended examples of selective in vitro inhibitors for CYP-mediated metabolism and respective quantitative data (K_i_/IC_50_) [47].

CYP Enzyme	Inhibitor	K_i_/IC_50_ (Μm) in In Vitro	References
CYP1A2	α-naphthoflavone	0.01	[166,167]
	Furafylline ^(1)^	0.6–0.7	[168,169,170,171]
CYP2B6	Clopidogrel ^(1)^	1.1	[172,173,174,175]
	Sertraline	3.2	[176,177]
	Thiotepa ^(1)^	2.8–3.8	[178,179,180,181,182]
	Ticlopidine ^(1)^	0.2–0.8	[172,173,177,180,183]
CYP2C8	Gemfibrozil glucuronide ^(1)^	52–75	[184,185,186,187]
	Montelukast	0.009–0.15	[188,189,190]
	Phenelzine ^(1)^	1.2	[187]
CYP2C9	Sulfaphenazole	0.3	[191,192,193]
	Tienilic acid ^(1)^	5	[194]
CYP2C19	N-3-benzyl-nirvanol	0.079–0.12	[195]
	Loratadine	0.76	[196]
	Nootkatone	0.5	[197]
	Ticlopidine ^(1)^	1.1	[198,199,200]
CYP2D6	Paroxetine ^(1)^	0.15	[201,202,203]
	Quinidine	0.018–0.06	[204,205,206]
CYP3A4	Azamulin ^(1)^	0.03–0.24	[207,208]
	Itraconazole	0.013–0.27	[191,209,210,211,212]
	Ketoconazole	0.0037–0.028	[173,191,193,210]
	Troleandomycin ^(1)^	0.26	[213,214,215]
	Verapamil ^(1)^	2.3–2.9	[216,217]

^(1)^ Time-dependent inhibitors.

**Table 5:** Examples of in vitro inducers for CYP-mediated metabolism as recommended by the FDA.

CYP Enzyme	Inducer	Class of Drugs	Mechanism (Receptor)
CYP1A2	Omeprazole	Proton pump inhibitors	AHR
CYP2B6	Phenobarbital	Barbiturates	CAR and PXR
CYP2C8	Rifampicin	Antibiotics	PXR
CYP2C9			
CYP2C19			
CYP2D6			
CYP3A4			

AHR, aryl hydrocarbon receptor; CAR, constitutive androstane receptor; PXR, pregnane X receptor.

**Table 6:** Selected examples of in silico studies for investigating DDIs.

Type of Study	Major Findings of the Study	Reference
PBPK model	A PBPK model was developed using in vitro, physicochemical, and clinical data to predict DDIs involving zanubrutinib. The model evaluated the effects of CYP3A inhibitors and inducers on zanubrutinib exposure, its impact on CYP3A4, CYP2C8, and CYP2B6 substrates, and the influence of gastric pH changes. This model was validated using clinical DDI data. It accurately predicted plasma concentrations and DDI outcomes.	[281]
PBPK model	This study aimed to predict the CYP3A-mediated DDI between saxagliptin and nicardipine using a PBPK model, incorporating in silico and in vitro data. PBPK models for both drugs were constructed using parameters derived from in vitro experiments and literature, and validated in rats, where co-administration resulted in 2.6-fold increase in saxagliptin exposure. The model was then extrapolated to humans, with simulations predicting only a minimal AUC increase (1.05-fold) indicating no clinically significant interaction. This study demonstrates the value of in vitro-informed PBPK modeling in assessing DDIs.	[282]
Deep learning method	The study presents a DDI prediction based on sequence and substructure features (SSF-DDI). By integrating these complementary data types, the model offers a more comprehensive molecular representation. Experimental results and case studies show that SSF-DDI significantly outperforms existing models, particularly in predicting DDIs involving previously unseen drugs, with a 5.67% improvement in accuracy over state-of-the-art approaches.	[283]
Quantitative structure-activity relationship (QSAR) model	QSAR models were developed using 11, 6, 10, 8, 8, 10, 10, and 10 substrates of CYP1A2, CYP2A6, CYP2B6, CYP2C9, CYP2C19, CYP2D6, CYP2E1, and CYP3A4, respectively.	[284]
ML method	The study introduces an ML framework for predicting DDI using simple rug target profile representations and an L2-regularized logistic regression model. This approach emphasizes biological interpretability by examining the gene-level relationships between drug targets. New statistical metrics are proposed to quantify DDI intensity, efficacy, and action range within protein–protein interaction (PPI) networks and signaling pathways. Empirical validation demonstrates the model outperforms existing models. Results reveal that DDIs are more likely when drugs share targets, their targets are closely connected in PPI networks, or they are involved in interacting pathways—offering mechanistic insights into potential adverse reactions.	[285]

## Data Availability

The original contributions presented in this study are included in the article. Further inquiries can be directed to the corresponding authors.
